# Supplementary material for: Drug repurposing for aging research using model organisms
Source: Aging Cell. 2017 Jun 16;16(5):1006–15. doi: 10.1111/acel.12626 (PMC5595691; doi:10.1111/acel.12626)
Supplement: Supplementary file 7 — Data S1 Zip‐Archive of all report cards. [file ACEL-16-1006-s007.zip › RC_TAK.pdf]

# TAK

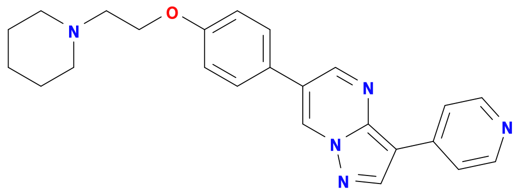

**Database identifiers**

| | |
|---|---|
| ChEMBLCompound | CHEMBL478629 |
| DrugBank | DB08597 |
| CHEBI | 78510 |
| eMolecules | 17282503 |

## Ranking

| | Rank | Score |
|---|---|---|
| Drosophila | 5/697 | 0.959 |
| C. elegans | 9/591 | 0.655 |

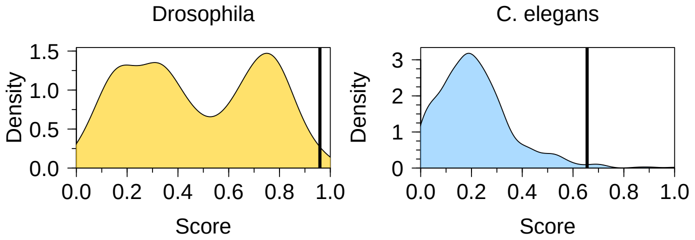


| | Ageing implication | Domain conservation | Binding site conservation | Binding affinity | Bioavailability | Lipinski | Promiscuity | Purchasability | Drug approval | **Total** |
|---|---|---|---|---|---|---|---|---|---|---|
| Drosophila | 1.0 | 0.969 | 1.0 | 0.9 | (0.9) | 0.0 | -0.0 | 0.1 | 0.075 | 0.959 |
| C. elegans | 1.0 | 0.965 | 0.923 | 0.9 | 0.6 | 0.0 | -0.0 | 0.1 | 0.075 | 0.655 |

## Names

- **dorsomorphin**
- 6-[4-(2-piperidin-1-ylethoxy)phenyl]-3-pyridin-4-ylpyrazolo[1,5-a]pyrimidine
- compound C

## Roles

EC 2.7.11.31 [hydroxymethylglutaryl-CoA reductase (NADPH)] kinase inhibitor, bone morphogenetic protein receptor antagonist

## Status

| | |
|---|---|
| Approved drug (according to ChEMBL) | No |
| Classification (according to DrugBank) | experimental |
| Number of Rule of 5 violations | 0 |
| Binding affinity to original target in log units (RF-Score prediction) | 7.2 |
| Burns *C. elegans* bioavailability prediction | 4.0 |

# Compound Target Characteristics

## 5'-AMP-activated protein kinase catalytic subunit alpha-2

Best gene implication in ageing for this target family came from gene Q95ZQ4 annotated in UniProt release 2014_02. Annotation GO 8340 (determination of adult lifespan) was Inferred from Mutant Phenotype

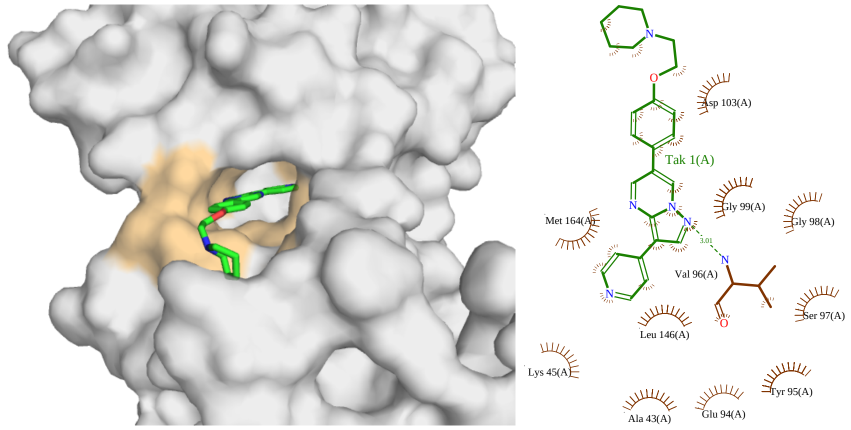


| protein | amino acids contacts (binding site) | | | | | | | | | | |
|---|---|---|---|---|---|---|---|---|---|---|---|
| PDB:3aqv:chainA:P54646 | A | K | E | Y | V | S | G | G | D | L | M |
| tr:Q96E92:Q96E92_HUMAN | A | K | E | Y | V | S | G | G | D | L | M |
| sp:P54646:AAPK2_HUMAN | A | K | E | Y | V | S | G | G | D | L | M |
| sp:Q13131:AAPK1_HUMAN | A | K | E | Y | V | S | G | G | D | L | M |
| tr:G3V715:G3V715_RAT | A | K | E | Y | V | S | G | G | D | L | M |
| sp:P54645:AAPK1_RAT | A | K | E | Y | V | S | G | G | D | L | M |
| sp:Q5EG47:AAPK1_MOUSE | A | K | E | Y | V | S | G | G | D | L | M |
| tr:Q3TUQ7:Q3TUQ7_MOUSE | A | K | E | Y | V | S | G | G | D | L | M |
| sp:Q8BRK8:AAPK2_MOUSE | A | K | E | Y | V | S | G | G | D | L | M |
| tr:Q5U118:Q5U118_DROME | A | K | E | Y | V | S | G | G | D | L | M |
| tr:O18645:O18645_DROME | A | K | E | Y | V | S | G | G | D | L | M |
| sp:Q95ZQ4:AAPK2_CAEEL | A | K | E | H | V | S | G | G | D | L | M |
| sp:P06782:SNF1_YEAST | A | K | E | Y | - | A | G | N | D | L | M |

| | whole protein | | domain-based | | contact-based | |
|---|---|---|---|---|---|---|
| protein | ident | simil | ident | simil | ident | simil |
| PDB:3aqv:chainA:P54646 | 1.0 | 1.0 | 1.0 | 1.0 | 1.0 | 1.0 |
| tr:Q96E92:Q96E92_HUMAN | 0.32 | 0.36 | 0.65 | 0.7 | 1.0 | 1.0 |
| sp:P54646:AAPK2_HUMAN | 1.0 | 1.0 | 1.0 | 1.0 | 1.0 | 1.0 |
| sp:Q13131:AAPK1_HUMAN | 0.74 | 0.9 | 0.91 | 0.97 | 1.0 | 1.0 |
| tr:G3V715:G3V715_RAT | 0.98 | 1.0 | 0.99 | 1.0 | 1.0 | 1.0 |
| sp:P54645:AAPK1_RAT | 0.74 | 0.89 | 0.91 | 0.97 | 1.0 | 1.0 |
| sp:Q5EG47:AAPK1_MOUSE | 0.74 | 0.89 | 0.9 | 0.97 | 1.0 | 1.0 |
| tr:Q3TUQ7:Q3TUQ7_MOUSE | 0.75 | 0.91 | 0.9 | 0.97 | 1.0 | 1.0 |
| sp:Q8BRK8:AAPK2_MOUSE | 0.98 | 0.99 | 0.99 | 1.0 | 1.0 | 1.0 |
| tr:Q5U118:Q5U118_DROME | 0.56 | 0.73 | 0.8 | 0.94 | 1.0 | 1.0 |
| tr:O18645:O18645_DROME | 0.56 | 0.73 | 0.8 | 0.94 | 1.0 | 1.0 |
| sp:Q95ZQ4:AAPK2_CAEEL | 0.51 | 0.71 | 0.76 | 0.93 | 0.91 | 0.92 |
| sp:P06782:SNF1_YEAST | 0.33 | 0.6 | 0.59 | 0.86 | 0.73 | 0.63 |

### SNF1A (FBgn0023169) associated phenotypes

RU486 conditional, cell cycle defective, cell polarity defective, cytokinesis defective, developmental rate defective, dominant, feeding behavior defective, hyperactive, immune response defective, increased cell size, lethal - all die before end of second instar larval stage, long lived, maternal effect, mitotic cell cycle defective, neuroanatomy defective, nutrition conditional, short lived, small body, somatic clone, some die during second instar larval stage, some die during third instar larval stage, starvation stress response defective, stress response defective

(Information from FlyBase)

**aak-2 (WBGene00020142) associated phenotypes**
drug induced gene expression variant, drug induced life span variant, drug induced locomotion variant, drug response variant, frequency body bend reduced, organism oxidative stress response hypersensitive, paraquat hypersensitive, protein phosphorylation reduced, suppression of head oscillations defective

(Information from WormBase)

**aak-2 (UniProt:Q95ZQ4) annotation**
**Function**: Acts as a sensor that couples lifespan to information about energy levels and insulin-like signals. Role in motility and response to oxidative stress. Causes germ cell cycle arrest upon dauer entry. (PubMed:15574588, PubMed:18408008).
**Enzyme regulation**: Activated by phosphorylation.(PubMed:15574588).
**Subunit**: Tetramer, composed of 2 regulatory (R) and 2 catalytic (C) subunits. In the presence of cAMP it dissociates into 2 active monomeric C subunits and an R dimer that binds four cAMP molecules. (PubMed:2303451).
**Tissue specificity**: Expressed in the pharynx, the ventral cord, neurons including the hermaphrodite-specific neuron, body wall muscles, the vulva, the excretory canal, and weakly in the intestine. (PubMed:18408008).
**Ptm**: Phosphorylated on Thr-243 in response to oxidative stress. (PubMed:15574588).
**Disruption phenotype**: Shortened life-span. Temperature-dependent hypersensitivity to stress, slow body bending, abnormal modulation of head oscillation. (PubMed:15574588, PubMed:18408008).

(Information from UniProt)
